# Interpretation of Antimicrobial Susceptibility Testing Using European Committee on Antimicrobial Susceptibility Testing (EUCAST) and Clinical and Laboratory Standards Institute (CLSI) Breakpoints: Analysis of Agreement

**DOI:** 10.7759/cureus.36977

**Published:** 2023-03-31

**Authors:** Priyanka Gaur, Vivek Hada, Rama S Rath, Aroop Mohanty, Parul Singh, Atul Rukadikar

**Affiliations:** 1 Microbiology, All India Institute of Medical Sciences, Gorakhpur, Gorakhpur, IND; 2 Community Medicine, All India Institute of Medical Sciences, Gorakhpur, Gorakhpur, IND

**Keywords:** fosfomycin, cefazolin, antimicrobial susceptibility testing, disk diffusion, amr, eucast, clsi

## Abstract

Objectives: Breakpoints provided by European Committee on Antimicrobial Susceptibility Testing (EUCAST) are now being used in many countries. This study was planned to ascertain the agreement in antimicrobial susceptibility using the Clinical and Laboratory Standards Institute (CLSI) and EUCAST breakpoints during the Kirby-Bauer disk diffusion method.

Methods: This was a prospective observational study. Clinical isolates belonging to the family *Enterobacteriaceae* recovered between January and December, 2022, were included in the analysis. The diameter of the zone of inhibition of the 14 antimicrobials (*viz*. amoxicillin/clavulanic acid, cefazolin, ceftriaxone, cefuroxime, cefixime, aztreonam, meropenem, gentamicin, amikacin, ciprofloxacin, levofloxacin, norfloxacin, trimethoprim/sulfamethoxazole and fosfomycin) was analysed. Antimicrobial susceptibility was interpreted using CLSI 2022 and EUCAST 2022 guidelines.

Results: Susceptibility data from a total of 356 isolates showed a slight increase in the percentage of resistant isolates with most of the drugs using EUCAST guidelines. The level of agreement varied from almost perfect to slight. For two drugs, i.e., fosfomycin and cefazolin, the agreement was least among the drug analysed (kappa (κ) value < 0.5, p < 0.001). For Ceftriaxone and Aztreonam, with EUCAST, susceptible (S) isolates would have been categorised in the newly redefined "I" category. It would have indicated the use of higher dosages of drugs.

Conclusion: Change in the breakpoints impacts the interpretation of the susceptibility. It can also lead to a change in the dosage of the drug used for treatment. Therefore, there is an urgent need to see the impact of recent modifications "I" category of EUCAST on the clinical outcome and usage of antimicrobials.

## Introduction

Antimicrobial susceptibility testing serves two essential functions: surveillance of antimicrobial resistance and detecting resistance in clinical isolates while treating patients. The method described by the Clinical Laboratory Standard Institute (CLSI) and its breakpoints are widely used methods of antimicrobial susceptibility testing [1]. In 1997, national agencies of various European countries formulated the European Committee on Antimicrobial Susceptibility Testing (EUCAST). It was initiated by the European Society of Clinical Microbiology and Infectious Diseases (ESCMID) to harmonize minimum inhibitory concentration (MIC) breakpoints across Europe [2]. As a result, almost all European countries have switched to EUCAST breakpoints for interpretation during antimicrobial susceptibility testing [3]. 

The World Health Organization recommends the methods described by CLSI and EUCAST for surveillance [4]. As per the worldwide norms, in India, the breakpoints provided by CLSI are used to interpret antimicrobial susceptibility. To maintain the comparability of data, the Indian Council of Medical Research also recommends using CLSI guidelines for surveillance [5].

In recent years, there has been an increase in the usage of breakpoints published by the EUCAST, and countries using EUCAST breakpoints have increased even outside the European continent [6]. The perceived advantages of EUCAST include transparent breakpoint setting processes, provision of rationale documents, absence of industry representatives on EUCAST committees, and the free availability of the documents [7]. However, limited studies have compared these guidelines’ impact on interpreting antimicrobial susceptibilities. Furthermore, most studies were completed before the significant change in the definition of susceptibility categories by EUCAST in 2019 [7]. These changes can significantly impact the results in laboratories adopting EUCAST breakpoints and dosage of antimicrobials for treating infections [8]. This study was thus aimed at assessing the level of agreement in the interpretation of antimicrobial susceptibility using these guidelines.

## Materials and methods

This observational study was conducted after approval from the Institutional Ethical Clearance Committee, All India Institute of Medical Sciences (AIIMS), Gorakhpur, India (approval number: IHEC/AIIMS-GKP/BMR/15/2020 dated December 25, 2020).

Antimicrobial susceptibility testing of clinical isolates using the Kirby Bauer Disk Diffusion method is done routinely in the clinical laboratory at AIIMS, Gorakhpur, India. Quality control was assured using quality control strains of *Escherichia coli *(ATCC 25922) and *Staphylococcus aureus* (ATCC 25923) as a routine practice. For this study, the zone of inhibition of microorganisms recovered in clinical samples (urine, blood, pus, sputum, etc.) during January and December, 2022, was recorded. The members of the *Enterobacteriaceae* family are the most isolated organisms in clinical microbiology laboratories; thus, only the organisms belonging to the family were included in the study. The identical isolates recovered from the repeat samples were excluded from the analysis. Drugs for which breakpoints for the zone of inhibition were available in both CLSI and EUCAST guidelines were included in the study. The antimicrobials with differences in disk content (piperacillin/tazobactam and cefotaxime) were excluded from the analysis. 

Interpretation of antimicrobial susceptibility for the antimicrobials included in the study was performed using CLSI 2022 and EUCAST 2022 breakpoints [9,10]. After measuring the zone of inhibition diameter, the isolates were categorised as per the respective guidelines. In both the Guidelines, Susceptible "S" and Resistant "R" are standard nomenclature. In CLSI breakpoints, "I" stands for Intermediate, and there is a separate category, "SDD", meaning Susceptible Dose-Dependent [9]. The meaning of “I” has been revised from "Intermediate" to "Susceptible Increased Exposure" in EUCAST. It implies a high likelihood of therapeutic success if exposure to the agent is increased by adjusting the dosing regimen or its concentration at the site of infection. In addition, a new category, "ATU", which means Area under Technical Uncertainty, has been incorporated in EUCAST. Due to technical challenges, the zone of inhibition labelled under ATU is difficult to interpret and is reported with caution [10,11]. All The data were entered in Microsoft Excel (Microsoft Corporation, Redmond, Washington, United States), and analysis was done in Stata Statistical Software: Release 12 (2011; StataCorp LP, College Station, Texas, United States) and Jamovi 2.3 (released 2022).

The extent of agreement between CLSI 2022 and EUCAST 2022 for various antimicrobials was evaluated using Cohen's Kappa statistics and graded from perfect agreement to poor agreement [12]. A p-value less than 0.05 was considered statistically significant for all inferential statistics.

## Results

During the study period, 356 nonrepeat isolates belonging to the family *Enterobacteriaceae* were recovered from various clinical samples. The most isolated organisms were *E coli* and *Klebsiella pneumoniae* (Table 1).

**Table 1:** Distribution of the microorganisms belonging to Enterobacteriaceae isolated in clinical samples during the study period

S No	Name of Organism	Urine	Pus	Sputum	Total
1.	Escherichia coli	187	25	6	218
2.	Klebsiella pneumoniae	72	22	16	110
3.	Klebsiella oxytoca	1	1	0	2
4.	Proteus mirabilis	1	11	1	13
5.	Proteus vulgaris	1	0	0	1
6.	*Enterobacter* spp	4	3	0	7
7.	*Morganella* spp	1	3	0	4
8.	Citrobacter	0	1	0	1
	Total	267	66	23	356

The zone of inhibition of the following 14 antimicrobials: amoxicillin/clavulanic acid, cefazolin, ceftriaxone, cefuroxime, cefixime, aztreonam, meropenem, gentamicin, amikacin, ciprofloxacin, levofloxacin, norfloxacin, trimethoprim/sulfamethoxazole and fosfomycin were compared based on breakpoints of CLSI and EUCAST guidelines. The antimicrobial to which *Enterobacteriaceae* were most susceptible was meropenem. The agreement was least for urinary antimicrobial cefazoline (κ = 0.3674) and fosfomycin (κ = 0.1261) (Table 2).

**Table 2:** Comparison of the percentage susceptibilities of the isolates along with measurement of agreement between antimicrobial susceptibility of the isolates using CLSI 2022 and EUCAST 2022 breakpoints. Interpretation of κ value, Almost perfect agreement: 0.81 to 0.99, Substantial agreement: 0.61 -0.80, Moderate agreement:0.41-60, Fair agreement: 0.21-1.40 Slight agreement: 0.01-0.20, Less than chance agreement: < 0. In EUCAST breakpoints, S: Susceptible, I: Susceptible Increased Exposure, R: Resistance, ATU: Area of Technical Uncertainty. In CLSI breakpoints, S: Susceptible, I: Intermediate, R: Resistant, SDD: Susceptible Dose Dependent. CLSI: Clinical and Laboratory Standards Institute; EUCAST: European Committee on Antimicrobial Susceptibility Testing Absolute numbers of the isolates are mentioned within ()

S No	Name of Antimicrobial	No. of Isolates	CLSI				EUCAST				Kappa (κ)	Agreement	p-value	Confidence Interval
			S	SDD	I	R	S	I	ATU	R				
1.	Amoxicillin/clavulanic Acid	331	32.9 (109)	-	18.4 (61)	48.6 (161)	21.1 (70)	-	-	78.9 (261)	0.4475	Moderate	<0.001	0.402 - 0.476
2.	Cefazolin	285	29.1 (83)	-	-	70.9 (202)	- (0)	23.9 (68)	-	76.1 (217)	0.3674	Fair	<0.001	0.248-0.487
3.	Ceftriaxone	355	33.8 (120)	-	3.7 (13)	62.5 (222)	24.8 (88)	11.3 (40)	-	63.9 (227)	0.7965	Substantial	<0.001	0.747-0.818
4.	Cefuroxime	142	13.4 (19)	-	20.4 (29)	66.2 (94)	26.1 (37)	(0)	-	73.9 (105)	0.5706	Moderate	<0.001	0.496-0.670
5.	Cefixime	273	28.9 (79)	-	5.1 (14)	65.9 (180)	33 (90)	(0)	-	67 (183)	0.8891	Almost perfect	<0.001	0.840-0.932
6.	Aztreonam	138	26.8 (37)	-	5.8 (8)	67.4 (93)	11.6 (16)	15.2 (21)	-	73.2 (101)	0.5499	Moderate	<0.001	0.453- 0.626
7.	Meropenem	144	76.4 (110)	-	4.2 (6)	19.4 (28)	77.8 (112)	7.6 (11)	-	14.6 (21)	0.833	Almost perfect	<0.001	0.801-0.929
8.	Gentamicin	187	68.4 (128)	-	2.7 (5)	28.9 (54)	55.6 (104)	-	-	44.4 (83)	0.6843	Substantial	<0.001	0.603-0.776
9.	Amikacin	181	57.5 (104)	-	15.5 (28)	27.1 (49)	40.9 (74)	-	-	59.1 (107)	0.4704	Moderate	<0.001	0.432- 0.547
10.	Ciprofloxacin	185	16.8 (31)	-	13 (24)	70.3 (130)	21.1 (39)	-	8.6 (16)	70.3 (130)	0.8706	Almost perfect	<0.001	0.808-0.932
11.	Levofloxacin	76	28.9 (22)	-	6.6 (5)	64.5 (49)	22.4 (17)	9.2 (7)	-	68.4 (52)	0.7843	Substantial	<0.001	0.561-0.858
12.	Norfloxacin	268	39.9 (107)	-	1.9 (5)	58.2 (156)	23.1 (62)	- (0)	-	76.9 (206)	0.5496	Moderate	<0.001	0.495-0.664
13.	Trimethoprim/sulfamethoxazole	200	53 (106)	-	3 (6)	44 (88)	54.5 (109)	- (0)	-	45.5 (91)	0.9413	Almost perfect	<0.001	0.887-0.972
14.	Fosfomycin	155	92.3 (143)	-	0.6 (1)	7.1 (11)	45.8 (71)	- (0)	-	54.2 (84)	0.1261	Slight	<0.001	0.065-0.232

**Figure 1:**
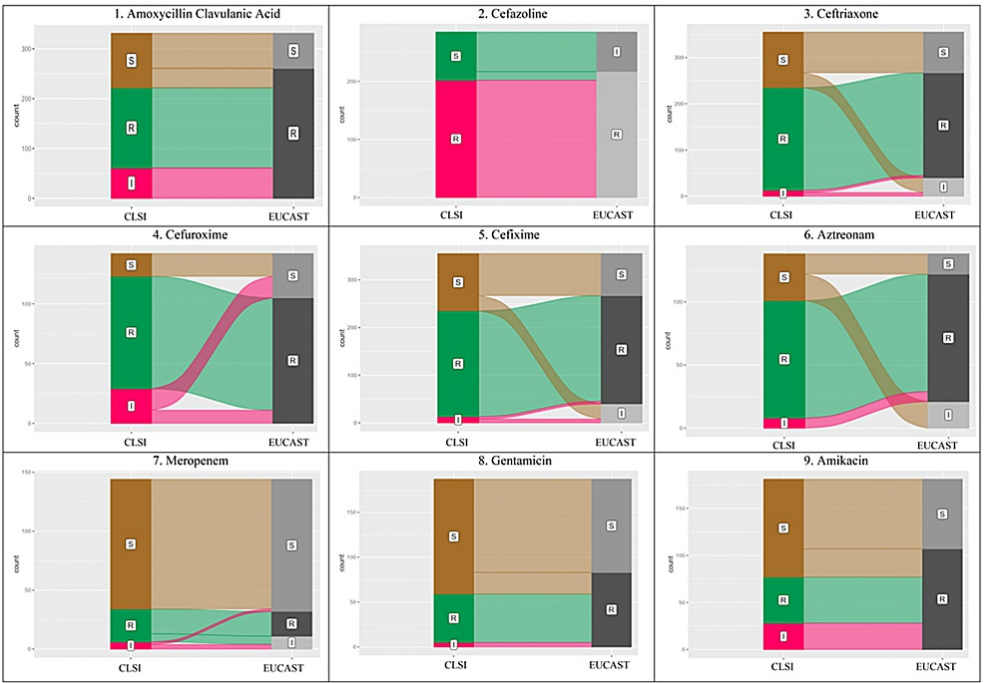
Alluvial plots showing change in interpretation using CLSI and EUCAST breakpoints of amoxycillin/clavulanic acid, cefazoline, ceftriaxone, cefuroxime, cefixime, aztreonam, meropenem, gentamicin, amikacin In EUCAST breakpoints, S: Susceptible, I: Susceptible Increased Exposure, R: Resistance, ATU: Area of Technical Uncertainty. In CLSI breakpoints, S: Susceptible, I: Intermediate, R: Resistant, SDD: Susceptible Dose Dependent. CLSI: Clinical and Laboratory Standards Institute; EUCAST: European Committee on Antimicrobial Susceptibility Testing

**Figure 2:**
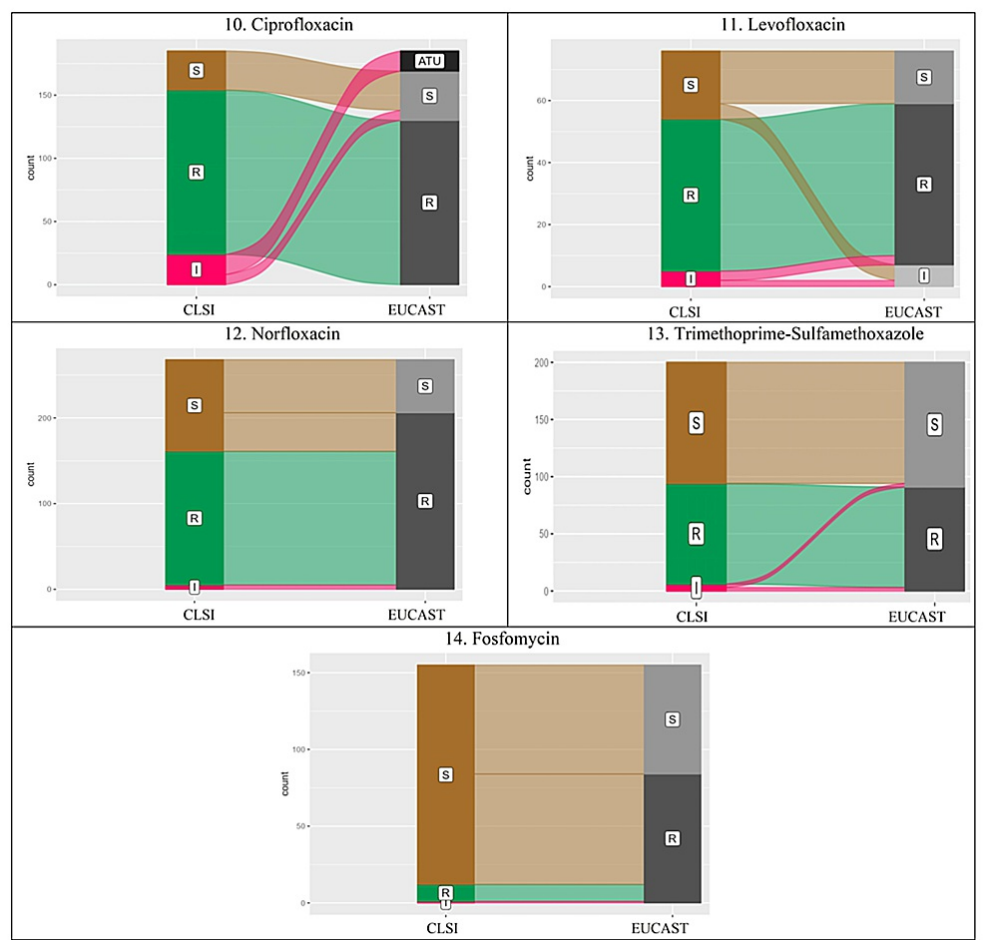
Alluvial plots showing change in interpretation using CLSI and EUCAST breakpoints of ciprofloxacin, levofloxacin, norfloxacin, trimethoprime- sulfamethoxazole, fosfomycin In EUCAST breakpoints, S: Susceptible, I: Susceptible Increased Exposure, R: Resistance, ATU: Area of Technical Uncertainty. In CLSI breakpoints, S: Susceptible, I: Intermediate, R: Resistant, SDD: Susceptible Dose Dependent. CLSI: Clinical and Laboratory Standards Institute; EUCAST: European Committee on Antimicrobial Susceptibility Testing

Almost perfect agreement (κ = 0.81-0.99) was observed in cefixime, ciprofloxacin, and meropenem. The percentage of resistant isolates increased with EUCAST breakpoints, except for carbapenems. The alluvial plots for various antimicrobials showed a change in interpretation in all the antimicrobials with the use of EUCAST breakpoints (Figures 1, 2). The isolates recognised as resistant using CLSI were also categorised as resistant using EUCAST breakpoints. But in other categories, changes were significant.

## Discussion

Antimicrobial resistance (AMR) is now considered a leading cause of death worldwide. Low resource settings have the highest burden of AMR. In 2019, six pathogens (E* coli, S. aureus, K pneumoniae*, *Streptococcus pneumoniae*, *Acinetobacter baumannii*, and *Pseudomonas aeruginosa*) were responsible for more than 250,000 deaths associated with AMR [13]. In the report published by AMR Research and Surveillance Network of the Indian Council of Medical Research, it was observed that *E coli *and *Klebsiella* spp were the most frequently isolated pathogen in the clinical samples in India. Also, there has been a significant decrease in the susceptibility to major antimicrobials among these two microorganisms [14]. In our study, *E coli *was the primary organism isolated from the cultures, followed by *K. pneumoniae*. 

The breakpoints for detecting drug susceptibility are critical; any alteration in the breakpoints can lead to interpretation changes. In addition, it can impact patient care, antimicrobial usage, and the comparison of surveillance data from various countries [7]. It has been noted in the past that EUCAST breakpoints are restrictive and tend to increase the resistance rates of microorganisms [1,15]. In our analysis of the antimicrobials, it was observed that the percentage of resistant isolates was more with EUCAST breakpoints as compared to CLSI except for meropenem. For amoxicillin/clavulanic acid, gentamicin, amikacin, and norfloxacin, the difference in the percentage of resistant isolates was more than 10%.

In this study for commonly used antimicrobial, amoxicillin/clavulanic acid, 16% of isolates were categorized as Intermediate. In these cases, increased dosage may provide therapeutic success per the definition of CLSI. However, in the EUCAST breakpoints, the category of Susceptible Increased Exposure for amoxicillin/clavulanic acid is not present for *Enterobacteriaceae*. Thus, all these isolates would have been categorized as resistant using EUCAST breakpoints (Figure 1). Therefore, it would have led to a change in the usage of the drug.

In the case of cefazoline, it was observed that agreement between the two guidelines was fair (κ = 0.3674, p < 0.005) and was mainly seen in the resistant category (Figure 1). Furthermore, with EUCAST breakpoints, not a single isolate was categorised as Susceptible. Instead, most of these isolates were classified as Susceptible to Increased Exposure. Thus, as per EUCAST guidelines in these cases, two grams thrice daily cefazoline is indicated for treatment as opposed to the dosage recommended by CLSI, i.e., one gram every 12 hours [4,6]. This interpretation discrepancy can impact the patient's dosage and thus affect the treatment outcome.

In susceptibility of aztreonam with EUCAST breakpoints, the Susceptible Increased Exposure (I) category would have 21% isolates and a dosage of two grams every six hours would have been indicated [10]. However, using CLSI guidelines, all these isolates were categorised as Susceptible with a recommended dosage of one gram every eight hours [9]. A similar phenomenon was observed in ceftriaxone, where isolates needed a higher dosage as per EUCAST (Figure 1).

In the case of meropenem, the percentage of the resistant isolate was slightly higher with CLSI (19.4 %) compared to EUCAST (14.6%). This was partly due to the availability of Susceptible Increased Exposure category in EUCAST with an option of higher dosage (two grams eight hourly over three hours), which contrasts with CLSI's recommended one standard dose (one gram administered eight hourly) [4,6]. Thus, in some cases, meropenem could have been indicated for treatment with a higher dosage (Figure 1). In a study by Swati et al. comparing the minimum inhibitory concentration (MIC) breakpoints of meropenem, similar observations were seen [15].

In the case of fosfomycin, the agreement was the least, and the percentage of susceptible isolates decreased by half while using the EUCAST guideline (Figure 2). It was majorly due to significant differences in the breakpoints of CLSI (S ≥ 16mm) and EUCAST (S ≥ 24mm). The agreement was moderate for another urinary antimicrobial, norfloxacin (κ = 0.3674, p<0.005). The susceptibility percentage decreased from almost 40% to 23% using EUCAST breakpoints. It was again mainly due to higher breakpoints of the zone of inhibition.

Guidelines used for interpretation can majorly impact the usage of antimicrobials for treating infections in all the above cases. The new definition of “I” in EUCAST aligns more with the SDD category in CLSI [16]. There is very restrictive use of the SDD category by CLSI in the currently available breakpoints. Among the drugs which were compared, breakpoints were not present in the SDD category for a single drug. Currently, there is a renewed focus on the correct usage of antimicrobials by implementing the antimicrobial stewardship program [17]. With the gradual implementation of the antimicrobial stewardship program, unequivocal interpretation of I will provide clear choices to clinicians while choosing the drugs for treatment. EUCAST aims to regenerate the I category's credibility. It may optimise and prolong the use of available antimicrobials with higher dosages. This will promote the use of available antimicrobials for the treatment of various infections [18].

All the AMR surveillance networks in India use CLSI breakpoints for detecting and reporting antimicrobial resistance [14,19]. Therefore, regular recording of the diameter of the zone of inhibition during disk diffusion in the laboratory can provide valuable input. Furthermore, they can be analysed simultaneously or retrospectively to assess the impact of EUCAST on surveillance data. 

One of the significant limitations of this study was the need for clinical correlation with therapeutic outcomes. Thus, future studies are needed to assess the overall impact of the switch between these guidelines on treatment outcomes, especially with increasing awareness of antimicrobial stewardship in India.

## Conclusions

In the disk diffusion method, the general agreement between the two guidelines varied from slight to almost perfect depending on the antimicrobial evaluated. However, as observed in the study using EUCAST, there was a significant change in the interpretation of antimicrobial susceptibility. Also, the difference in the meaning of the I category in EUCAST guidelines would have impacted the usage of antimicrobials compared to CLSI guidelines. The use of an increased dosage of antimicrobials for category I drug in EUCAST would prevent unnecessary usage of reserved antimicrobials. This is the cornerstone for promoting rational usage of antimicrobials and slowing down the emergence of drug resistance. Therefore, there is a need to evaluate the effect of these disagreements on treatment outcomes in the patient as well as the usage of antimicrobials. 
